# Psychological Impact on Maxillofacial Trauma Patients – An Observational Study

**DOI:** 10.25122/jml-2020-0111

**Published:** 2020

**Authors:** Jay Kishore, Ritesh Vatsa, Jyotirmay Singh, Minti Kumari, Tanoj Kumar, Subhash Bandgar

**Affiliations:** 1. Bihar Government, Primary Health Center, Sahdei Buzurg, Vaishali, Bihar, India; 2. Department of Dentistry, Sri Krishna Medical College and Hospital, Muzaffarpur, Bihar, India; 3. Department of Orthodontics and Dentofacial Orthopaedics, Patna Dental College and Hospital, Patna, Bihar, India; 4. Department of Public Health Dentistry, Patna Dental College and Hospital, Patna, Bihar, India; 5. Department of Oral Pathology, Patna Dental College and Hospital, Patna, Bihar, India; 6. Department of Prosthodontics, Nair Hospital Dental College, Mumbai, Maharashtra, India

**Keywords:** Hospital Anxiety and Depression Scale (HADS), Maxillofacial trauma, psychological implications, post-traumatic stress, Trauma Screening Questionnaire (TSQ)

## Abstract

Facial disfigurement due to trauma is very common as the face is a prominent part of the body and is susceptible to injuries. A protocol for the diagnosis and intervention for psychological problems of trauma patients should be implemented in the Indian hospitals as they lack policies for assessing the mental status of such patients. This study was conducted to analyze and determine the psychological implications and need for mental health services of maxillofacial trauma patients. Fifty patients with maxillofacial trauma above 18 years of age were included in this study. The assessment was done using the Hospital Anxiety and Depression Scale (HADS) and Trauma Screening Questionnaire (TSQ), which are considered as the standard tools for assessment of psychological disorders post-trauma. We observed psychological stress in 84% of the patients at the baseline, which reduced to 24% after one month at the first follow-up visit and further reduced to 22% at the second follow-up visit. The reduction in the percentage from the first to the second follow-up visit was less significant. However, the change in HADS and TSQ scores was found to be significant, suggesting that the intensity of trauma decreases with the time-lapse. Anxiety, depression, and post-traumatic stress are prevalent in patients with maxillofacial trauma, which may lead to impaired day-to-day life. Our results suggest that there is a need for psychological care in patients with maxillofacial trauma.

## Introduction

Facial disfigurement due to trauma is very common as the face is a prominent part of the body and is susceptible to injuries. Trauma can be due to road traffic accidents, sports injuries, assaults, falls, and other work-related injuries. Apart from the mortality, this type of trauma causes psychological stress, depression, anxiety are other post-traumatic disorders which affect the patient.

Facial disfigurement post-trauma may lead to depression and anxiety and delay the healing process [1]. Trauma to the face may lead to disfigurement; a scar on the face is traumatizing, and facial disfigurements are the least desirable impairments and can become a stigma. Complete recovery of individuals with this type of trauma depends on multiple factors of medical, social, personal and psychological nature. Hence, during the treatment of patients with maxillofacial trauma, it is imperative to address psychological issues other than the surgical treatment to improve patients’ quality of life.

Usually, maxillofacial traumas are non-lethal, but they can cause major psychological problems, including anxiety, depression, and post-traumatic stress disorder. However, these problems are usually ignored and remain untreated due to the lack of diagnosis [5, 6].

Psychological care is an essential factor for traumatized patients; still, Indian hospitals lack policies for assessing the mental status of post-trauma patients.

Post-traumatic stress disorder (PTSD) following any traumatic event is defined as patients suffering from symptoms like a feeling of danger, nervousness, vulnerability, and repeated memories of the event, persisting more than 30 days after the traumatic incident [7]. Depression has also been associated with PTSD.

The importance of diagnosis and intervention for psychological problems in the patients following trauma has been noted in many studies. These authors also showed that regular interventions in such patients resulted in a decrease in long-term negative effects on the patient’s complete health [8, 9].

Previous studies indicated that anxiety and depression in patients with maxillofacial trauma showed an uphill trend in the initial 6 months post-trauma period and decreased later. Our study was conducted to analyze the psychological implications of maxillofacial trauma patients. This study will help in determining the need for mental health services in maxillofacial trauma victims.

There are many tools for the assessment of psychological disorders. Out of many, the Hospital Anxiety and Depression Scale (HADS) with the anxiety and depression subscales, each having 7 questions, measures anxiety and depression using a 0-to-21 scale where a score greater than 7 indicates a disorder. Another tool, the Trauma Screening Questionnaire (TSQ), was used by many studies to assess the post-traumatic stress disorder.

## Material and Methods

This study was conducted on patients with maxillofacial trauma reporting to the routine out-patient and emergency departments.

Patients with maxillofacial trauma above 18 years of age were included in this study. Patients with life-threatening injuries like brain injury, orthopedics injury, chest injury, abdominal injury, and vascular injury were excluded from the study.

Maxillofacial trauma patients were included in the study after follow-up visits at 1 week, 1 month, and 6 months. The assessment was done using HADS and TSQ, which are considered as the standard tools for the assessment of psychological disorders post-trauma; along with the above-mentioned tools, socio-demographic and clinical data will also be reported.

The Hospital Anxiety and Depression Scale consists of a 14-point self-report questionnaire with anxiety and depression subscales. Every point is marked on a 4-point scale (0-3), with each subscale ranging from 0 to 21. A total score above threshold (7) is indicative of disorder [2]. For post-traumatic stress disorder, we took a trauma screening questionnaire [3], which is a 10-point scale and each point is derived from the DSM-IV criteria found in the Diagnostic and Statistical Manual of Mental Disorders [4]. This scale defines symptoms of re-experience of PTSD, which includes items 1-5 and arousal symptoms of PTSD, including items 6-10. A total score of 6 or more was considered as a diagnostic of post-traumatic stress disorder.

A total of 80 patients were selected for the study, but only 50 patients of them gave consent to participate in the study. Out of those 50 patients, three groups were made according to age. Group I included patients in the 18-30 years category, group II included patients in the 31-45 category, while group III included patients that were older than 45 years. Patients below 18 years were not included in the study.

### Hospital Anxiety and Depression Scale

It consists of a 14-item scale, which is a self-report instrument, including anxiety and depression subscales. Each item is marked on a 4-point scale (0-3), each subscale ranging from 0 to 21. A score above threshold (7) indicates a disorder [2].

### Trauma Screening Questionnaire (TSQ)

It is a symptom-based screening tool for post-traumatic stress disorder. It consists of a scale with 10 items, each of which is derived from the DSM-IV criteria [4] describing two symptoms, one of which is

a re-experiencing symptom of PTSD, which includes items 1 to 5 and the other one including arousal symptoms of PTSD (items 6 to 10). A score of 6 or more is diagnostic of post-traumatic stress disorders.

Statistical analysis was done using the Chi-square test, and the percentage is used for the demographic and clinical data.

## Results

Table 1 shows that patients involved in trauma are more frequently males compared to females. In our study, we could recruit only 18 females as compared to 32 males. 48% of the cases recruited fell into the first group, suggesting that patients suffering from injuries are younger, the number subsequently decreasing with increasing age.

**Table 1:** Distribution of male and female patients into groups according to age.

Sex (N=50)	Group I (n=24)	Group II (n=19)	Group III (n=7)
Male (32)	15 (30%)	13 (26%)	4 (8%)
Female (18)	9 (18%)	6 (12%)	3 (6%)
Percentage	48%	(38%)	14%

Table 2 shows the change of the HADS value from the baseline (10 days post-trauma) to the first follow-up (1 month post-trauma) and second follow-up (6 months post-trauma). It was observed that the change in the HADS score is significant at the first and second follow-ups; however, the increase in the change of score is greater at the second follow-up compared to the first follow-up. When the first and second follow-up visits were compared, it was noted that the score was significantly lower at the second follow-up, suggesting that the psychological depression is greater during the first month, and it decreases as the time elapses.

**Table 2:** Depressive disorder change at baseline, first follow-up and second follow-up (HADS).

	First follow-up (1 month)		Second follow-up (6 month)	
	Score ≤7	Score >7	Score ≤7	Score >7
**Base Line Score** ≤**7**	31	2	30	1
Base Line Score >7	2	12	8	7
Chi-Squared test	0.000		0.000	

Table 3 compares the HADS scores in patients with a soft-tissue injury and those with maxillofacial fractures at the baseline. It was observed that patients with soft tissue trauma had significantly less anxiety as compared to maxillofacial fractures, which suggests that facial appearance is a more significant concern in trauma patients.

**Table 3:** Comparison of anxiety (HADS) in soft-tissue injury and maxillofacial fracture patients at baseline.

	HADS anxiety ≤7 (baseline visit)	HADS anxiety of 8 or more (baseline visit)
Soft-tissue injuries	25	2
Maxillofacial trauma	13	10
Chi-squared test (Significant at p <0.05)	P=0.0029	

Table 4 shows the TSQ scores in soft-tissue injury patients are lower as compared to maxillofacial fracture patients, suggesting that maxillofacial fracture patients are at significantly more stress compared to the soft-tissue injury patients.

**Table 4:** TSQ score in soft-tissue injury and maxillofacial fracture patients at baseline.

	TSQ ≤6 (baseline visit)	TSQ ≥7 (baseline visit)
Soft-tissue injuries	24	2
Maxillofacial fractures	14	10
Chi-squared test (Significant at p < 0.05)	P=0.00495	

The change in the TSQ scores from baseline to the first follow-up was noted in all patients. Regarding TSQ scores, the baseline was 1 month. Table 5 suggests that the change from first to second follow-up in the TSQ score is significant. Therefore, the HADS scores suggest that anxiety, depression and post-traumatic stress disorder are reduced as time elapses and is highest from day 10 to one month; it subsequently decreases along with the healing process.

**Table 5:** Change in the TSQ score from the first to the second follow-up visit.

	TSQ ≤5 (Second follow-up at 6 months)	TSQ > 5 (Second follow-up at 6 months)
TSQ ≤5 (First follow-up at 1 month)	33	1
TSQ > 5 (First follow-up at 1 month)	3	10
Chi-Squared test	0.000	

## Discussion

Our study recruited 50 patients with maxillofacial trauma to ascertain the psychological disorders after trauma. Out of 50 patients, 32 were males and 18 females, suggesting that males are more prone to injuries in India as they are the ones more involved in outdoor activities in the Indian scenario. Baseline readings for these patients were performed 10 days post-trauma, keeping in mind the life-threatening condition of the patient, and only those patients who were not in a life-threatening state were included in the study. The first follow-up was scheduled at 1month post-trauma and the second after 6 months post-trauma.

In our study, patients with soft-tissue injury were more numerous compared to maxillofacial trauma. However, it was not statistically significant. The number of females was lower, which could be due to the greater mobility attributed to males compared to females. Another factor that was observed in this study that more patients were on two-wheelers as compared to the four-wheelers and the number of patients wearing helmets was very low, suggesting that road safety measures reduce fatal accidents and other comorbidities associated with road traffic accidents if followed strictly. Our results are consistent with the studies conducted by Bansod S *et al*. [10], Jayadevan *et al*. [11], and Sharma *et al*. [12].

Behavioral changes were also observed in our study in post-trauma patients, which included anxiety while driving or traveling. These changes were also observed by Mayou *et al*. [13]. In the current study, out of 50 subjects, 18 (36% of the cases) showed behavioral changes post-trauma. These changes can hamper routine life activities.

We also evaluated the pattern of injury and took into account the soft-tissue injuries in which patients had laceration, avulsion, abrasion and contusion. In total, we noted soft-tissue injuries in 52% of the cases.

Maxillofacial fracture in the patients of our study included multiple and isolated facial bone fractures, where the majority had multiple fractures. Mandibular parasymphysial fractures were the most common, and there was only one case of isolated parasymphysial fracture; the rest were multiple. Among the isolated fractures, zygomatic, Le Fort II and III fractures were observed.

Studies from India reported that zygomatic fractures are the most common midfacial fracture, and the mandible is the most common in the lower-third; in all cases, parasymphysial fractures were found to be the most common [14, 15].

We studied the psychological stress disorder in post-trauma patients by using HADS and TSQ. We observed psychological stress in 84% of the patients at the baseline, which reduced to 24% after 1 month (the first follow-up visit) and further reduced to 22% at the second follow-up visit. The reduction in the percentage of patients from the first to the second follow-up visit was less significant. However, the change in the HADS and TSQ scores was found to be significant, suggesting that the intensity of trauma decreases as time elapses. Our results were found to be consistent with the study conducted by Ukpong *et al*. [16]; The authors found that 90% of their cases had psychological stress at baseline.

The anxiety and depression were studied using HADS, and it was observed that there was a significant reduction of anxiety and depression from baseline to the first follow-up and from baseline to the second follow-up.

TSQ was used as a tool for assessing PTSD. It was found that there was a significant reduction of the score at the second follow-up compared to the first follow-up visit, suggesting that post-trauma stress decreased significantly from month 1 (first follow-up) to month 6 (second follow-up).

We also observed that anxiety, depression, and post-traumatic stress disorder were more commonly present in patients with maxillofacial trauma than soft-tissue injury patients.

## Conclusion

Anxiety, depression, and post-traumatic stress are very common in maxillofacial trauma patients, which may lead to an impaired day-to-day life. Although the symptoms disappear as time elapses, it is still important to address physiological disorders in trauma patients in order to help them fully recover physically and psychologically.

## Conflict of Interest

The authors declare that there is no conflict of interest.
